# A case report of largest rectal prolapse in the literature successfully treated with Altemeier's procedure

**DOI:** 10.1016/j.amsu.2022.104231

**Published:** 2022-07-31

**Authors:** Mojtaba Ahmadinejad, Yeganeh Farsi, Mohammad hadi bahr, Ramin bozorgmehr, Ali soltanian, Javad zebarjadi bagherpour

**Affiliations:** a Department of Surgery, Shahid Madani Hospital, Alborz University of Medical Sciences, Alborz, Iran; b Student Research Committee, Shahid Beheshti University of Medical Sciences, Tehran, Iran; c Department of Surgery, Alborz University of Medical Sciences, Alborz, Iran

**Keywords:** Rectal prolapse, Altemeier's procedure

## Abstract

**Background:**

The rectal prolapse is defined as the concentric protrusion of full or partial thickness of the rectum or rectosigmoid via the anus. This is an increasing clinical concern that is usually found in old female patients.

**Cases presentation:**

A 39-year-old male patient was referred due to an un-reduceable rectal projection from a week ago. The primary endeavor for reduction of the projection under sedation and after local mannitol treatment at the operation room was unsuccessful, so surgical resection and reduction were planned for the patient.

**Conclusion:**

Management of rectal prolapse has always been one of the challenges of colorectal surgery. For patients with incarcerated prolapse manual reduction under sedation is used. If the reduction is unsuccessful, surgical procedures are used.

## Highlights

• The rectal prolapse is defined as the concentric protrusion of full or partial thickness of the rectum or rectosigmoid via the anus.
• Management of rectal prolapse has always been one of the challenges of colorectal surgery.
• Giant rectal prolapse defined as segment of rectosigmoid larger than 10 cm in length.

## 1 Introduction

The rectal prolapse is defined as the concentric protrusion of full or partial thickness of the rectum or rectosigmoid via the anus and this condition is estimated to occur in less than 0.5% of the population [1]. Complete rectal prolapse, also known as rectal procidentia or the third-degree prolapse is the full thickness projection, is an increasing clinical concern that is usually found in old female patients [2,3]. Increased intra-abdominal pressure in conditions such as chronic constipation, or pelvic floor/anal sphincter musculature weakness is the main underlying condition [2,4]. Once the projection occurs, nonoperative or operative interventions are mandatory to reduce it as soon as possible with the least complications [5]. In patients with acute prolapse who present to the emergency department, non-surgical methods are initially used for reduction. If the reduction is unsuccessful, surgical procedures are used. Several surgical procedures have been defined to treat prolapse the choice of surgical procedure depends mainly on the patient's age, clinical condition, surgical risk, coexisting functional symptoms, and the familiarity of the surgeon with a particular surgical approach. Based on Surgical Case Report, 2020 (SCARE) guidelines [6], In this article, we report a young man with a huge complete rectal prolapse who presented to our emergency department.

## 2 Case presentation of rectal projection

**Fig. 1:**
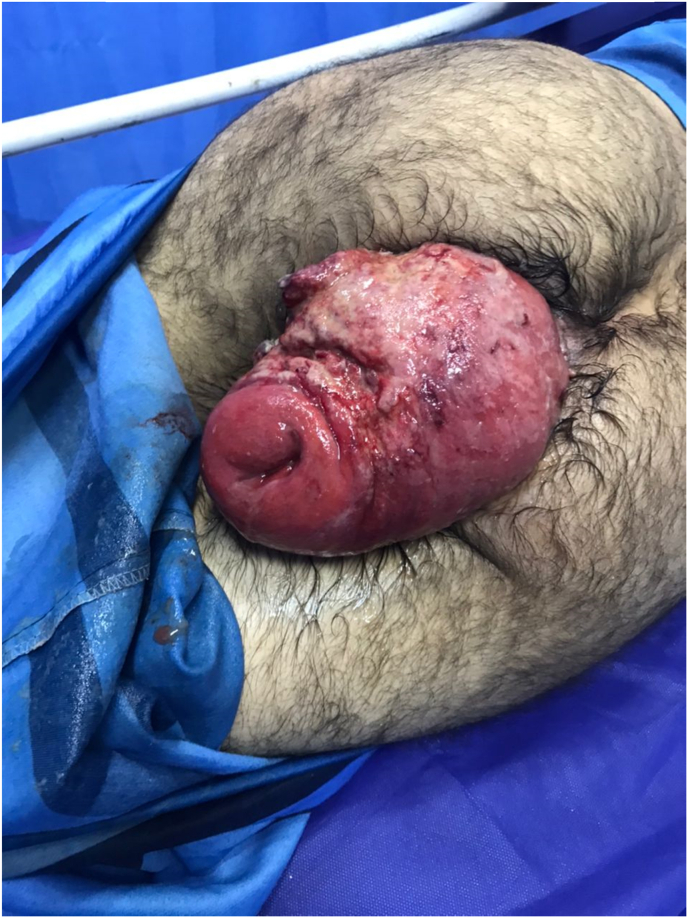
Large rectal prolapse.

**Fig. 2:**
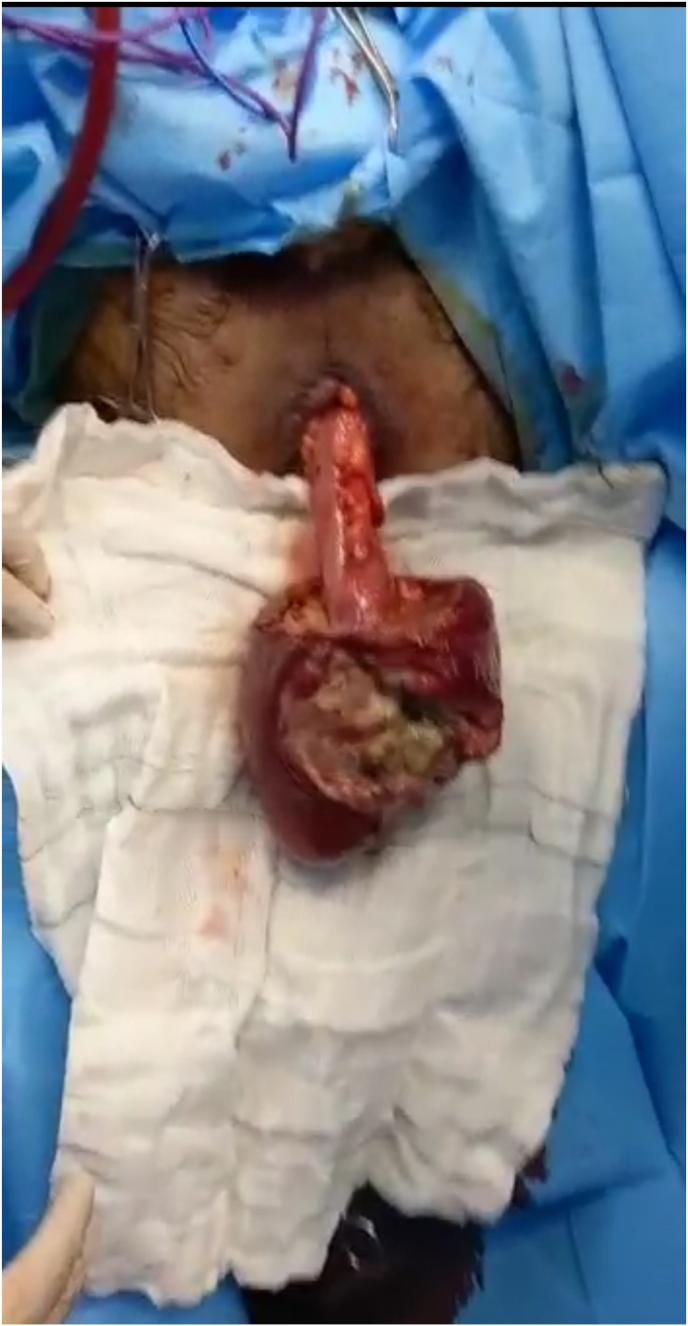
Rectum before resection.

**Fig. 3:**
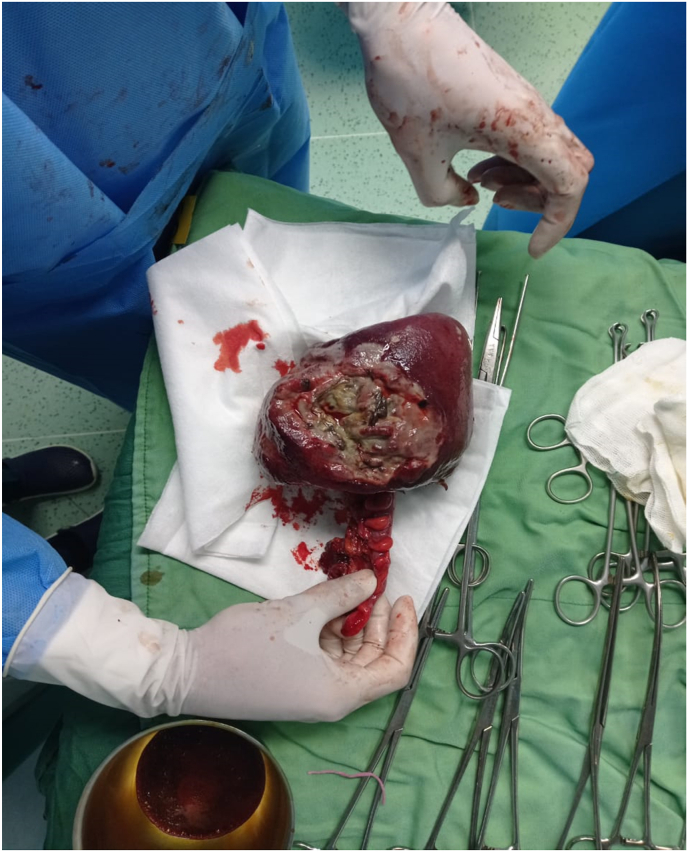
Resected rectum.

**Fig. 4:**
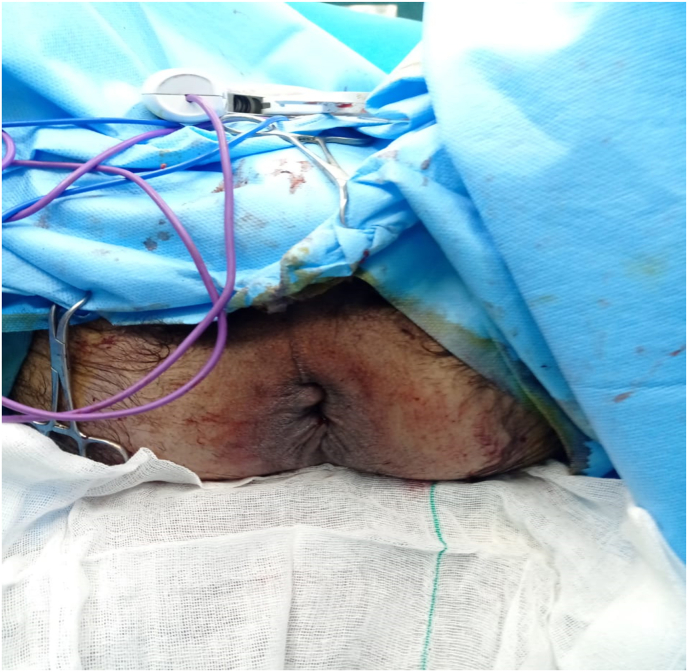
After coloanal anastomosis.

A 39-year-old male patient was referred to the surgery department of Shahid Madani Hospital in Alborz province in Iran via ambulance due to an un-reduceable rectal projection. The patient had a history of chronic constipation and opium addiction. Also, he noted previous episodes of rectal projection which were reduced to their origin via manipulation. The patient had a negative drug and other medical histories. The current state has been initiated a week ago and progressed till referring. Physical examination revealed an awake and oriented patient, which was not ill or toxic. He was not feverish and did not note any nausea or vomiting. The gas pass and defecation were not impaired. Full-thickness projection of the rectum, with an ulcerative surface and purulent secretions, was notable (Fig. 1). The primary endeavor for reduction of the projection under sedation and after local mannitol treatment at the operation room was unsuccessful, so surgical resection and reduction were planned for the patient. Before surgery, the patient underwent a total colonoscopy, which was normal. In the operation room with the patient in lithotomy position and under general anesthesia, a circumferential incision was made with electrocautery on the entire rectal wall two cm from the dentate line (Fig. 2, Fig. 3). Following ligature of the mesorectum and the mesosigmoid, the prolapsed rectosigmoid was resected and an anastomosis, interrupted by hand in two planes, was created (Fig. 4). Then in the supine position with an incision in the RLQ, an area double ileostomy ostomy was constructed by the attending surgeon to protect the anastomosis. The post-operative period was uneventful and the patient was discharged 5 days after surgery. The pathological examination of the resected segments revealed no abnormalities but local inflammation. Manometry of the anal sphincter showed decreased pressure at rest. The patient underwent six biofeedback sessions after which anal manometry showed that the anal sphincter had a normal tone at rest. The patient was hospitalized 6 months after surgery and the ileostomy was closed without complication. During an outpatient follow-up visit for 6 months, there was no problem and was satisfied with the type of treatment he received.

## 3 Discussion

Our patient was a young man with a history of opium addiction and chronic constipation for years which full-thickness rectal projection brought him to the emergency department. Due to primary failure in reducing the rectal prolapse and incarceration, the patient was a candidate for surgical management; altemeier rectosigmoidectomy with an ileal diversional loop.

According to the latest version of the World Society of Emergency Surgery and the American Association for the Surgery of Trauma (WSES- AAST) guideline, the management of uncomplicated, non-strangulated cases of rectal prolapse, should be initiated with non-operative procedures including submucosal epinephrine or hyaluronidase administration, rectal insertion of hypertonic dextrose or mannitol treated gauze, and elastic band wrapping, in a mildly sedated patient with Trendelenburg position [7]; as we have performed in our patient. In strangulated cases, complicated patients with hemodynamic instability, or evidence of peritonitis, gangrene, and perforation, surgical intervention should be considered as soon as possible which can be performed via either perineal or abdominal approaches, open or laparoscopically [[7], [8], [9]]. It is known that there are more than 100 operative procedures for the treatment of rectal prolapse. the operative procedure for rectal prolapse should be individualized according to each patient's background and degree of rectal prolapse. Perineal procedures are reported to have lower morbidity and mortality but higher recurrence rates are also noted [8,10]. In a study conducted by Hu et al., 51 male patients were followed after surgical management of rectal prolapse via abdominal or perineal approaches; overall, lower rates of complications and recurrence were reported in the abdominal approach group but the constipation was improved more notably in the perineal approached group [11]. Since the abdominal approach is difficult to adopt, we had no choice but to select the perineal approach in this case. Perineal proctosigmoidectomy, also known as Altemeier's procedure is one of the popular techniques in the emergency setting for almost all patients with acceptable outcomes [4,12,13]. Also, a temporary diversional loop such as ileostomy or colostomy can be considered for patients to ensure the excellence of anastomosis healing due to the surgeon's judgments [14].

## 4 Conclusion

In-time surgical management after primary failure in the non-operative management of complete rectal prolapse warrants an acceptable clinical course with minimum perioperative complications and excellent short time surgical outcomes.

## Ethical approval

This is a case report paper.

## Sources of funding for your research

There was no funding in this research.

## Author contribution

All authors had same contribution on this work.

## Conflicts of interest

There are no conflicts of interest.

## Registration of research studies

1. Name of the registry:
2. Unique Identifying number or registration ID:
3. Hyperlink to your specific registration (must be publicly accessible and will be checked):

## Guarantor

The corresponding author is Dr. Javad zebarjadi bagherpour who accepts full responsibility for the work and/or the conduct of the study, had access to the data, and controlled the decision to publish.

## Consent

Informed consent was obtained from the patient for publication of this case report and accompanying images. A copy of the written consent is available for review by the Editor-in-Chief of this journal on request.

## Provenance and peer review

Not commissioned, externally peer-reviewed.
